# Supplementary material for: New Solid Forms of Nitrofurantoin and 4-Aminopyridine Salt: Influence of Salt Hydration Level on Crystal Packing and Physicochemical Properties
Source: Molecules. 2022 Dec 16;27(24):8990. doi: 10.3390/molecules27248990 (PMC9783863; doi:10.3390/molecules27248990)
Supplement: Supplementary file 1 [file molecules-27-08990-s001.zip › molecules-2096251-supplementary.pdf]

Supporting Materials

for

New Solid Forms of Nitrofurantoin and 4-aminopyridine Salt: Influence of Salt Hydration Level on Crystal Packing and Physicochemical Properties

Boycov D.E.,[1] *Drozd K.V.,*[1] *Manin A.N.,*[1] *Churakov A.V.,*[2] *Perlovich G.L.*[1*]

[1]G.A. Krestov Institute of Solution Chemistry of the Russian Academy of Sciences, 1 Akademicheskaya St., Ivanovo, 153045, Russian Federation

[2]Institute of General and Inorganic Chemistry, Russian Academy of Sciences, 31 Leninsky Prosp., Moscow, 119991, Russian Federation

* Corresponding author: glp@isc-ras.ru

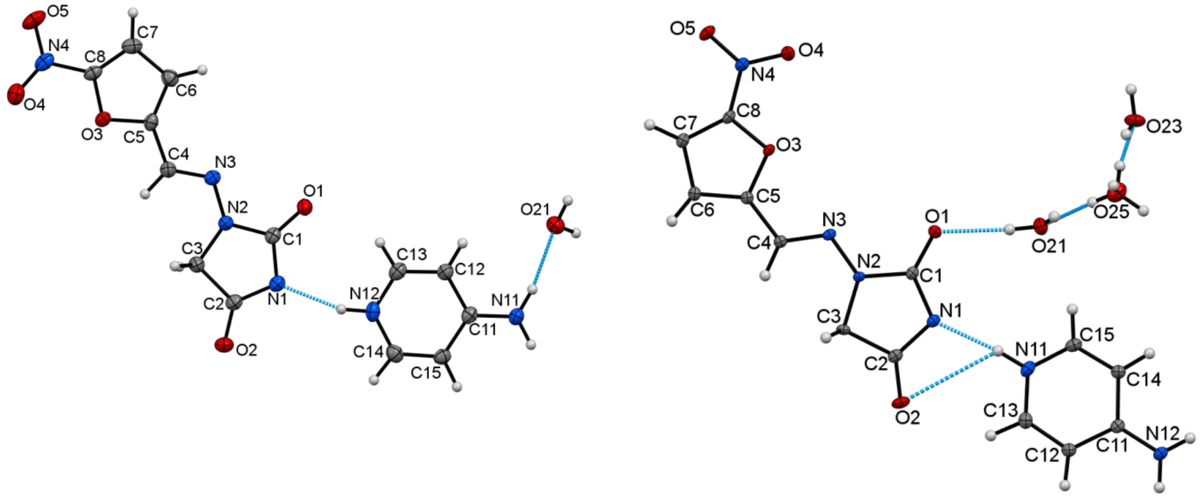

(a) (b)

Figure S1. The ORTEP diagram of (a) [NFT+4AmPy+H2O] salt (1:1:1) and (b) [NFT+4AmPy+H2O] salt (1:1:4). Thermal ellipsoids are drawn at the 50% probability level. Intermolecular interactions are drawn as blue dashed lines.

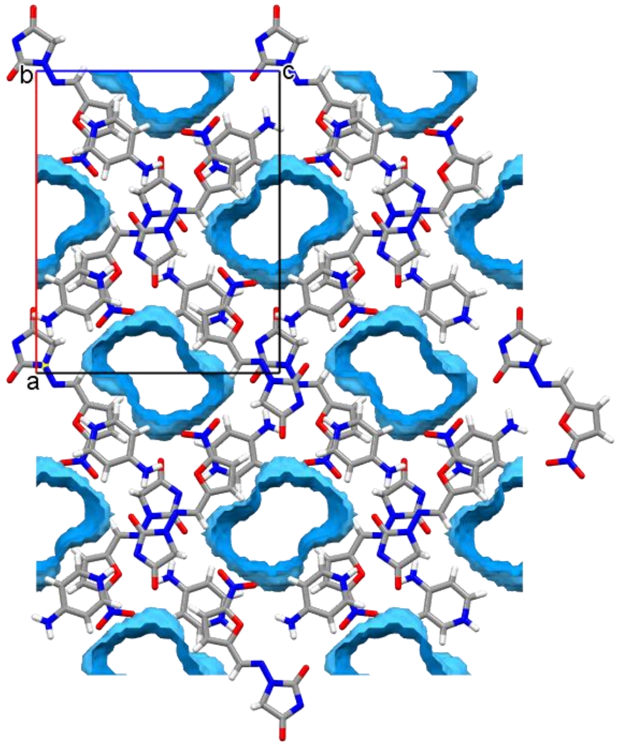

Figure S2. Water interaction map for the [NFT+4AmPy+H2O] salt (1:1:4).

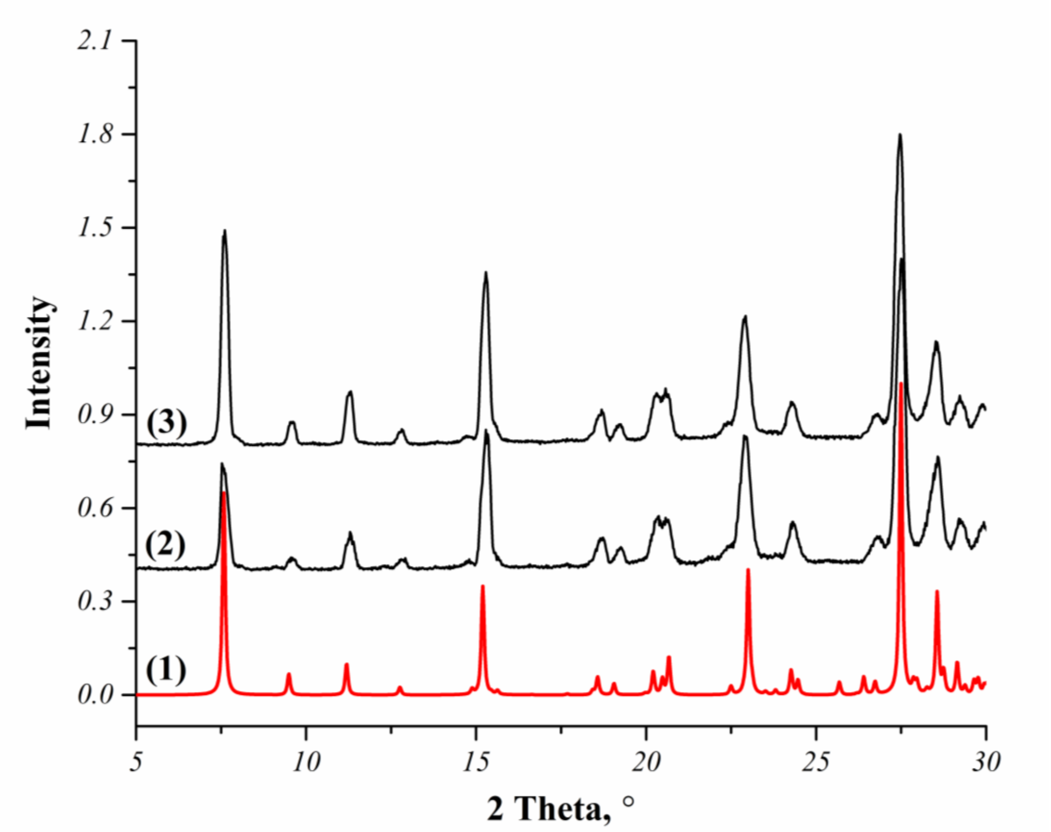

Figure S3. Comparison of the simulated PXRD calculated for the [NFT+4AmPy+H_2O] salt (1:1:4) (1) with the experimental PXRD patterns of the powder samples obtained by LAG of the [NFT+4AmPy] salt (1:1) (2) and the [NFT+4AmPy+H_2O] salt (1:1:1) (3) in the presence of water.

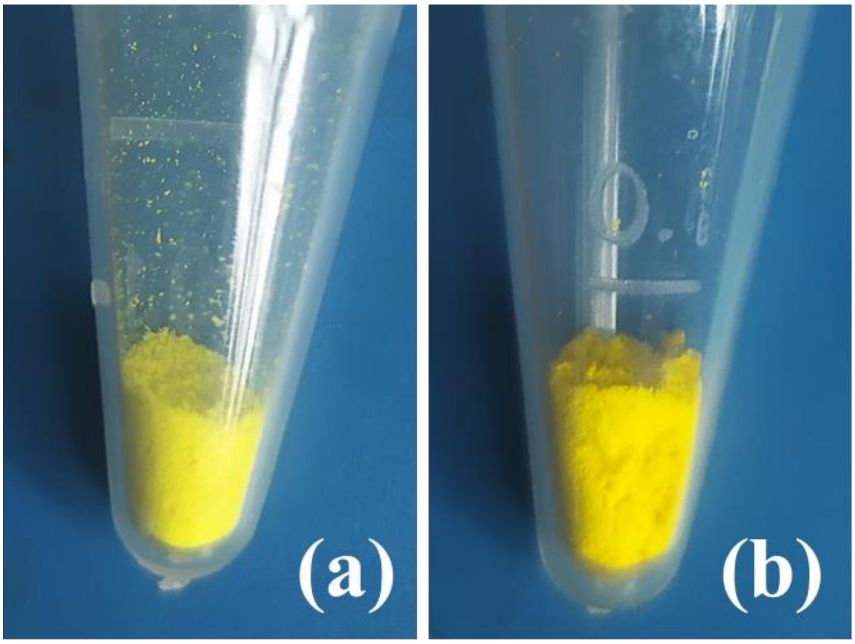

Figure S4. Color difference between (a) [NFT+4AmPy+H_2O] salt (1:1:1) and (b) [NFT+4AmPy+H_2O] salt (1:1:4) prepared by LAG method.

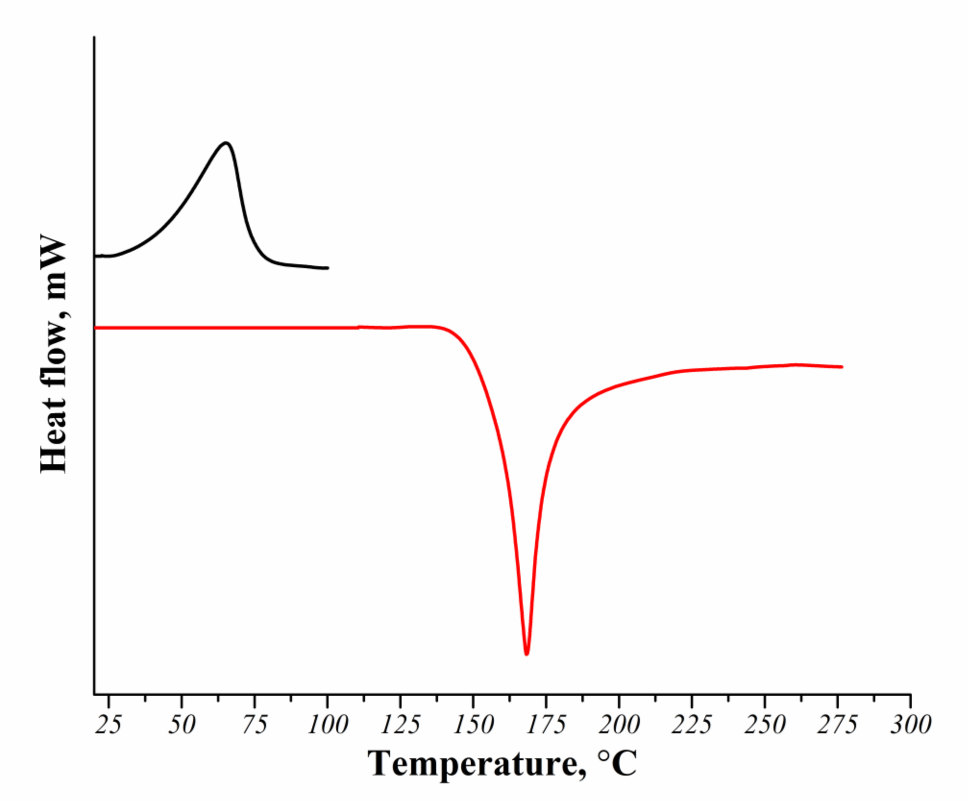

(a)

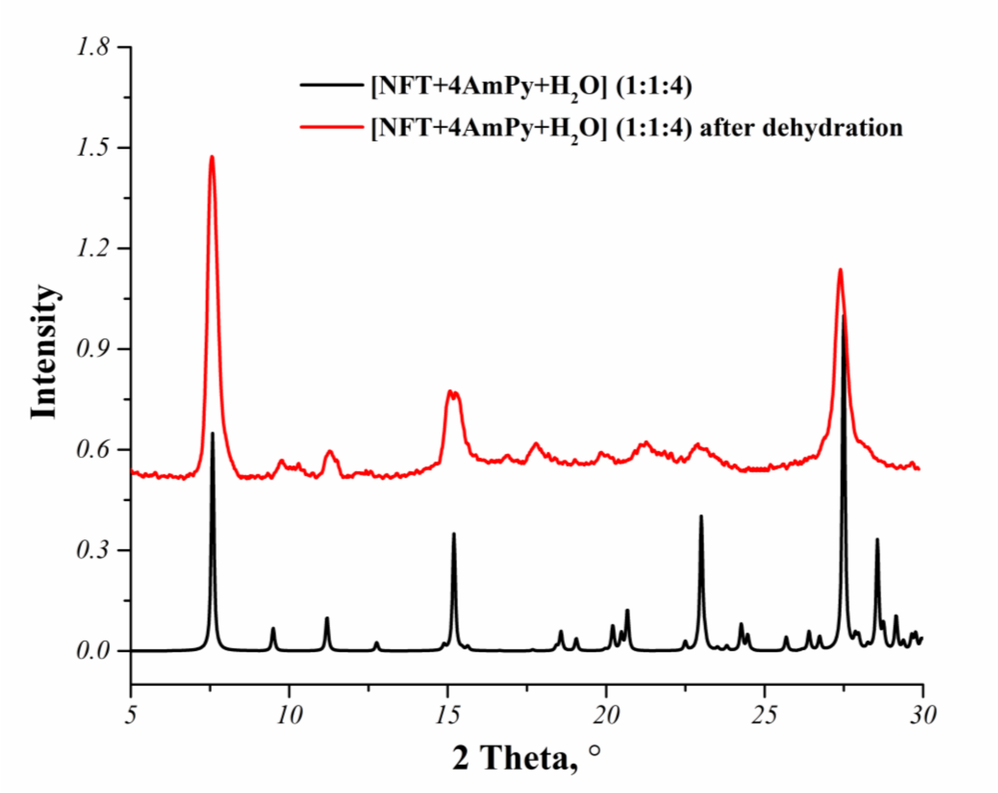

(b)

Figure S5. (a) DSC curves of the [NFT+4AmPy+H_2O] salt (1:1:4): the black line shows the first heating, the red one – the second heating; (b) comparison of the experimental PXRD pattern of [NFT+4AmPy+H_2O] salt (1:1:4) after dehydration and the simulated PXRD pattern calculated from the SCXRD data.

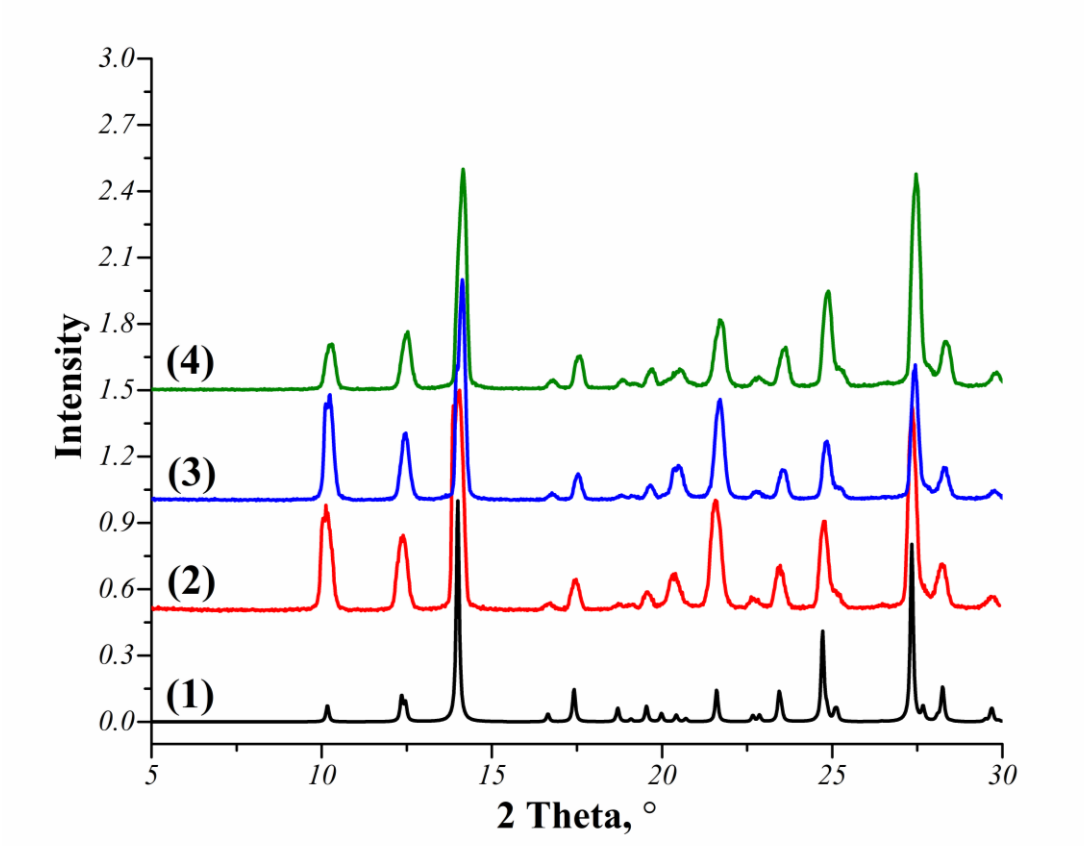

Figure S6. Results of PXRD analysis of the residual materials collected at the end of the dissolution experiments: (1) calculated PXRD patterns of NFT monohydrate; (2) [NFT+4AmPy] (1:1); (3) [NFT+4AmPy+H_2O] (1:1:1) and (4) [NFT+4AmPy+H_2O] (1:1:4).

Table S1. Hydrogen bond geometries for the NFT hydrated salts

D—H···*A*	*D*—H, Å	H···*A*, Å	*D*···*A*, Å	*D*—*H*···*A*, °
**[NFT+4AmPy+H_2O] salt (1:1:1)**				
$N12^+$—H1···$N1^-$	0.904 (19)	1.963 (19)	2.8396 (17)	162.9 (16)
N11—H10···O21	0.91 (2)	2.03 (2)	2.9241 (18)	167.7 (17)
N11—H11···$O1^{i}$	0.905 (19)	2.030 (19)	2.8973 (17)	160.2 (16)
O21—H20···$O2^{ii}$	0.90 (2)	1.88 (2)	2.7669 (16)	169 (2)
O21—H21···$O2^{iii}$	0.87 (2)	1.97 (2)	2.8286 (17)	169 (2)
Symmetry codes: (i) *x*+1/2, −*y*+1/2, *z*−1/2; (ii) *x*+1/2, −*y*+1/2, *z*+1/2; (iii) −*x*+3/2, *y*−1/2, −*z*+1/2				
**[NFT+4AmPy+H_2O] salt (1:1:4)**				
$N11^+$—H1···$N1^-$	0.91 (3)	1.86 (3)	2.7669 (18)	174 (3)
O21—H21···O1	0.82 (3)	1.94 (3)	2.7645 (18)	176 (3)
O21—H22···O25	0.75 (4)	2.08 (4)	2.7837 (15)	156 (4)
O23—H24···$O2^{i}$	0.82 (4)	1.93 (4)	2.7523 (18)	178 (3)
O23—H23···O25	0.81 (4)	1.99 (4)	2.7496 (15)	156 (4)
O25—H28···O21	0.78 (4)	2.02 (4)	2.7837 (15)	166 (3)
O25—H25···O23	0.81 (4)	1.99 (4)	2.7496 (15)	158 (3)
O25—H26···$O25^{ii}$	0.86 (3)	1.94 (3)	2.793 (2)	172 (3)
O25—H27···$O25^{iii}$	0.84 (4)	1.95 (4)	2.754 (2)	160 (4)
N12—H10···$O3^{iv}$	0.87 (2)	2.36 (2)	3.1269 (17)	148 (2)
N12—H11···$O1^{iv}$	0.89 (2)	2.09 (3)	2.9628 (18)	168 (2)
Symmetry codes: (i) x+1/2, y, −z+3/2; (ii) −x+1, −y+2, −z+2; (iii) x, −y+5/2, z; (iv) x−1/2, y, −z+3/2				
